# Protective effects of *Trifolium alexandrinum* L. against lung injury induced by environmental toxin CCl_4_ in experimental rats

**DOI:** 10.3402/fnr.v60.30433

**Published:** 2016-11-07

**Authors:** Rahmat Ali Khan, Huda Mohammad Alkreathy, Abdus Saboorshah, Mushtaq Ahmed, Samiullah Khan

**Affiliations:** 1 Department of Biotechnology, Faculty of Biological Sciences, University of Science and Technology Bannu, Khyber Pakhtunkhwa, Pakistan; 2 Department of Pharmacology, King Abdulaziz University(KAU), Jeddah, Saudi Arabia; 3 Department of Microbiology, Faculty of Biological Sciences, Quaid-i-Azam University, Islamabad, Pakistan

**Keywords:** lungs, CCl_4_, lipid peroxidation, *Trifolium alexandrinum*

## Abstract

**Background:**

In Pakistan numerous medicinal floras has used in the treatment of various human ailments. Among them *Trifolium alexandrinum* L. is traditionally used in the curing of disease. Presently we designed to ascertain the protective role of *Trifolium alexandrinum* methanolic extracts (TAME) against carbon tetrachloride (CCl_4_)-induced lung injury and oxidative stress in rats.

**Methods:**

Exposure to CCl_4_ induces oxidative stress and causes tissue damage by the induction of CCl_4_ free radicals. Twenty-four male albino rats were divided equally into four groups. Rats in group I had free access to drinking water and laboratory food. Group II was treated with 1 ml/kg body weight (b.w.) CCl_4_ (30% in olive oil). Groups III and IV rats were fed (p.o.) 200 mg/kg b.w. TAME and 50 mg/kg b.w. silymarin after 24 h of CCl_4_ treatment for 2 weeks.

**Results:**

Administration of CCl_4_ caused a significant (*p*<0.01) decrease in the activities of antioxidant enzymes (catalase, peroxidase, glutathione peroxidase, glutathione-*S*-transferase), and glutathione contents were decreased; however, thiobarbituric acid-reactive substances were increased (*p*<0.01). The alterations caused by CCl_4_ were significantly (*p*<0.01) reversed toward control levels by supplementation of TAME and silymarin.

**Conclusion:**

These results suggest that in rats TAME and silymarin could protect the lungs against CCl_4_-induced oxidative damage.

The epithelium cells of the lungs are frequently exposed to the highest concentration of oxygen and gaseous exchanges that make them susceptible to pulmonary toxicity (1). Pulmonary toxicity is caused by the generation of ROS (highly reactive oxygen species) that alter the structure of protein, nucleic acid, and other macro molecules (2). During aerobic metabolism, in normal situations, the toxic metabolites of oxygen are formed in low quantity in lung cells (3, 4). This normal production of ROS performs an essential role in a number of physiological activities but, in excess, the generation of ROS embraces several ailments (5). Pulmonary diseases like asthma, hyperoxia, and sarcoidosis are believed to be linked with oxidative stress. Chronic pulmonary disease (CPD) is the foremost cause of death, both in the industrialized and developing countries. It is associated with a progressive decrease in lung function that ultimately results in emphysema and chronic bronchitis (6). Different environmental factors like sunlight, UV light exposure, smoking, and chemicals are the main causes of the generation of ROS (7). Carbon tetrachloride (CCl_4_) is a synthetic industrial chemical that induces hepatic and renal toxicity in workers as well as in experimental animals (8) via ingestion, skin absorption or intraperitoneal intoxication, or by the inhalation of CCL_4_ lipid peroxidation, which increases while the antioxidant defense enzymes and total tissue proteins decreases (9). CCl_4_ is metabolized into trichloromethyl radical (^•^CCl_3_) that combines with oxygen and forms a more reactive peroxy trichloromethyl radical (^•^OOCCl_3_). These free radicals produce alkoxy (R•) and peroxy radicals (ROO•) that form lipid peroxides, and cause enzymatic inactivity and tissue injury (10). CCl_4_ also decreases tissue proteins and the antioxidant enzymes like SOD, peroxidase (POD), and catalase (CAT) (11, 12). Oxidative stress initiates and continues a range of lung, liver, and kidney damages. Therefore, it is of the utmost importance to evaluate agents with powerful antioxidant potential against the attack of CCl_4_ (13). The majority of foodstuff and natural products have been reported to be excellent means of chemo-protection. *Curcuma longa* has been reported to have therapeutic activity due to its antioxidant potential (14, 15). Similarly, the methanolic extract of *Ephorbia prostrata* is a potential phototoxic and antioxidant that markedly inhibits free radicals due to the presence of flavonoids and polyphenolic compounds (16). The phytochemical assessment of *Trifolium alexandrinum* has revealed the presence of steroids, terpenoinds, flavonoids, and isoflavonoids (17). Hence it was selected for the present study for the evaluation of the capability of *T. alexandrinum* to reduce the toxicity induced by CCl_4_ in the lungs of albino rats.

## Materials and methods

### Chemicals

Methanol, glutathione reduced (GSH), glutathione oxidized (GSSG), glutathione peroxidase (GSH-Px), glutathione-*S*-transferase (GST), bovine serum albumin (BSA), 5,5-dithio-bis-(2-nitrobenzoic acid) (DTNB), 1-chloro-2,4-dinitrobenzene (CDNB), reduced nicotinamide adenine dinucleotide phosphate (NADPH), CCl_4_, thiobarbituric acid (TBA), trichloroacetic acid (TCA), Folin–Ciocalteu reagent, sodium hydroxide, disodium hydrogen phosphate, sodium dihydrogen phosphate, and DMSO of Sigma Aldrich Chemicals Co. were utilized in this experiment.

### Plant preparation and extraction

During the maturity period, *T. alexandrinum* (L.) plants (leaves, stem, and flowers) grown in the north region of Bannu District, Pakistan, were collected. Then it was taxonomically identified by Prof. Abdur Rahman, serving in the Department of Botany, the Post Graduate College of Bannu. The plant extract was chopped and dried under shade for a period of one month. Then, after drying, the chopped plant was grinded into 1-mm fine powder with the help of a mechanical grinder. The powdered material (700 g) was soaked in 2.5 liter (80%) methanol for a period of 1 week at room temperature and then filtered. The filtrate was collected and concentrated with the help of a rotary evaporator at 38°C, under reduced pressure, and the crude extract was stored at 4°C for further assessment.

### Animals and treatment

The study protocol was approved by the ethical committee of University of Science and Technology of Bannu, for laboratory and experimental animal feed and care. The present experiment was designed with 24 adult albino rats. The rats, weighing 200–210 g, were divided equally into four groups; kept under normal laboratory conditions on 12 h light/darkness, at room temperature; and fed standard feed and water. Group I was the control and remained untreated, while group II was treated with 1 ml/kg body weight (b.w.; 30% in olive oil i.p.) CCl_4_. Group III received 50 mg/kg b.w. silymarin, and group IV was treated with 200 mg/kg b.w. *T. alexandrinum* methanolic extracts (TAME) after 24 h of CCl_4_ treatment. At the end of the experiment, after 24 h of the last dose, the rats were weighed and sacrificed. Lungs were removed, washed with saline, then dried with filter paper, and weighed. Lung tissues were stored at −70°C for further enzymatic analysis.

### Assessment of protein, CAT, and POD

Homogenized lung tissue (200 mg) was placed in four volumes of 0.1 mM NaH_2_PO_4_ buffer (pH 7.4) and centrifuged at 6,000×g for 30 min. The supernatant was collected and used for the analysis of antioxidant enzymes and protein. Protein was determined in the lung homogenate by the method of Lowry et al. (18), using BSA as standard. The activities of CAT and POD were carried out by the protocol of Chance and Maehly (19). In brief, the CAT activity was examined using hydrogen peroxide (H_2_O_2_). Change in absorbance was noted at 240 nm. The activity of POD was determined at 470 nm by the use of guaiacol as a substrate. One unit of enzymes (CAT and POD) was defined as an absorbance change of 0.01 as units/min.

### GST assay

GST activity was determined by the method of Habig et al. (20), using CDNB as a substrate. GST was measured at 340 nm using a molar extinction coefficient of 9.6×10^3^ M^−1^ cm^−1^.

### GSH-Px assay

An assay of GSH-Px was determined by the method of Mohandas et al. (21). NADPH was used as a substrate and the absorbance measured at 340 nm. The molar coefficient was 6.22×10^3^ M^−1^ cm^−1^.

### Glutathione reductase activity

Activity of glutathione reductase in the lungs homogenate of rats was determined by the protocol of Carlberg and Mannervik (22), with some modifications. The change in absorbance was measured at 340 nm. NADPH was used as a substrate. An extinction coefficient of 6.22×10^3^ M^−1^ cm^−1^ was used for calculations.

### Estimation of TBARS

Thiobarbituric acid–reactive substances (TBARS) in lungs homogenate were estimated by the TBA method described by Wright et al. (23). Change in absorbance was noted at 535 nm. An extinction coefficient of 1.56×10^5^ M^−1^ cm^−1^ was used for TBARS calculation.

## Results

Results of this study have shown that CCl_4_ toxicity significantly (*p*<0.01) reduced the antioxidant enzyme level of the lung compared to the control group (Table 1). The co-treatment of 50 mg/kg b.w. silymarin and TAME at doses of 200 mg/kg b.w. significantly (*p*<0.01) restored the activities of CAT and POD in the lungs of rats. TAME at the dose of 200 mg/kg b.w. restored completely the CAT and POD activities closely toward control level.

**Table 1:** Effect of TAME on the protein and lung marker enzymes of rats

Treatment	Protein (µg/mg tissue)	CAT (U/min)	POD (U/min)
Control	280.7±9.6++	1.7±0.4++	3.1±0.5++
CCl_4_	187.4±13.2**	0.9±0.2**	1.7±0.3**
CCl_4_+silymarin	277.8±10.6++	1.5±0.3++	3.0±0.6++
CCl_4_+TAME	230.5±12.3++	1.1±0.1++	2.7±0.5++

Mean±SEM (*n*=6).

** Indicate significance from the control group at *p*<0.01 probability level.

++ Indicate significance from the CCl_4_ group at *p*<0.01 probability level.

The activities of GST and GSH-Px in the lungs of rats were significantly (*p*<0.01) decreased by intoxication of CCl_4_ that were partially reversed by the supplementation of 200 mg/kg b.w. TAME. The results shown by silymarin group also effectively (*p*<0.01) reduced the impact of CCl_4_ on these considerations as shown in Table 2.

**Table 2:** Effect of TAME on GSH-Px and GST in rat lungs

Treatment	GST (nmol/min/mg protein)	GSH (mol/g tissue)	GSH-Px (mol/g tissue)
Control	34.9±3.2++		110.8±5.3++
CCl_4_	23.3±2.5**	13.93±1.4++	99.7±4.8**
CCl_4_+silymarin	34.5±2.7++	8.14±0.9**	112.2±6.3++
CCl_4_+TAME	28.7±3.1++	13.78±1.3++	108.1±5.7++

Mean±SEM (*n*=6).

** Indicate significance from the control group at *p*<0.01 probability level.

++ Indicate significance from the CCl_4_ group at *p*<0.01 probability level.

**Fig. 1:**
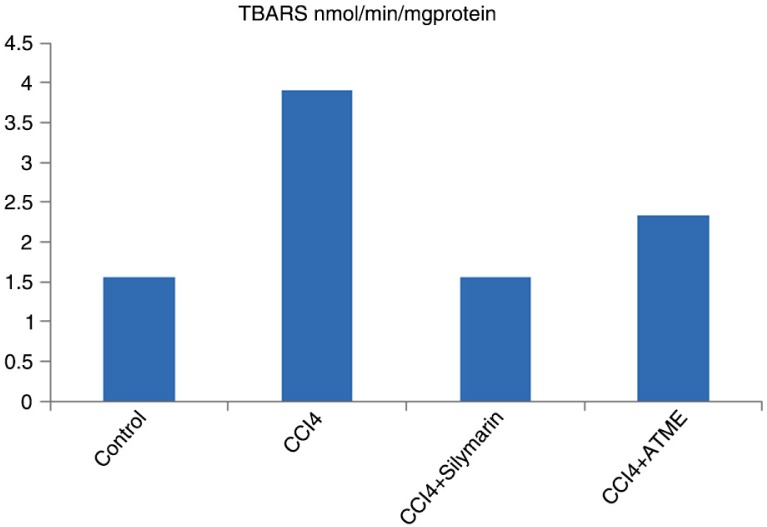
Effects of TAME on TBARS in rat lungs.

The contents of GSH in the lungs were significantly (*p*<0.01) decreased while the TBARS contents increased in the CCl_4_ intoxicated group as shown in Table 2 and Fig. 1, respectively. The altered level of GSH and TBARS by CCl_4_ was reduced by the oral addition of methanolic extract of *T. alexandrinum* (L.). Silymarin also reduced the CCl_4_-induced perturbations in the above markers.

The protective role of *T. alexandrinum* (L.) against CCl_4_ intoxication in rat on lungs weight and relative lungs weight and body weight are shown in Table 3. CCl_4_ significantly increased (*p*<0.01) lung weight, relative lung weight, and body weight as compared to control group treatment of 200 mg/kg b.w. *T. alexandrinum* (L.) concentrations ameliorated the CCl_4_ toxicity and reduced (*p*<0.01) the lung weight, relative lung weight, significantly like that of standard silymarin.

**Table 3:** Effect of TAME on the body and lung weight of rats

Treatment	Initial body wt (g)	Final body wt (g)	% increase body wt	Absolute lungs wt	Relative lungs wt
Control	190.3±5.7++	198.2±5.2++	4.2±0.7++	1.5±0.5++	0.75±0.05++
CCl_4_	185.2±6.2**	192.3±9.0**	3.8±0.5**	1.9±0.2**	0.98±0.07**
CCl_4_+silymarin	185.7±6.1++	195.8±7.8++	5.4±0.9++	1.5±0.4++	0.76±0.04++
CCl_4_+TAME	185.4±7.3++	194.6±5.7++	4.8±0.5++	1.5±0.3++	0.77±0.09++

Mean±SEM (*n*=6).

** Indicate significance from the control group at *p*<0.01 probability level.

++ Indicate significance from the CCl_4_ group at *p*<0.01 probability level.

## Discussion

In this experiment, the recovery potential of the TAME was taken under consideration against CCl_4_ toxicity in the lungs of rats. The main observations in these experiments were the capability of TAME to recover the inflammatory-oxidant activity of CCl_4_. It is reported that CCl_4_ induced toxicity due to its conversion to CCl_3_, which is a highly reactive free radical. These CCl_3_ free radicals damage DNA and lipids, and contribute to the loss in protein and enzyme activity (24). Results shown in Table 3 have revealed that CCl_4_ intoxication in the rats significantly lowers the percent increase in body weight. The absolute weights of the rat lungs and the relative lung weights increased as compared to those of the control rats in the CCl_4_ group. Minimal increase in the body weights may be due to the degeneration of body tissue while the increase in the lung weights may be the effect of the inflammatory response of the rat's lungs, as was evidenced in another study (25). Supplementations of TAME and silymarin possibly change the response and restore the changes in body and lung weights to those closer to the weights of the control group. CCl_4_ intoxication significantly reduces the activities of CAT, and POD in lungs, which might be due to a depletion of GSH contents (26). The low level of GST and GSH-Px correlates to oxidative stress and the depletion of GSH in the lungs resulting from the treatment of CCl_4_ administered to the rats. Normally, GSH is converted into GSSG, which is then reduced by glutathione reductase to GSH. TBARS is a highly reactive aldehyde formed as a result of the peroxidation of polyunsaturated fatty acids. TBARS formation is considered a sign of tissue damages (27). The high level of TBARS accumulation in the lungs, as a result of CCl_4_ treatment, indicates the extensive oxidative damage to lung tissues. The TAME extract contains polyphenols and flavonoids which have antioxidant and anti-inflammatory roles and reduce the risk of oxidative ailments. The reduction in the level of lipid peroxidation and the improved activity of antioxidant enzymes proved that TAME and silymarin are capable of protecting against various pathological conditions, including oxidative stress (28).

## Conclusion

In rats, TAME significantly protects the lungs against oxidative damages, which might be due to the presence of bioactive constituents in the extract.
